# Sodium-glucose Co-transporter 2 Inhibitors in Acute Heart Failure: A Review of the Available Evidence and Practical Guidance on Clinical Use

**DOI:** 10.31083/j.rcm2304139

**Published:** 2022-04-11

**Authors:** Herminio Morillas, Emilio Galcerá, Edgardo Alania, Julia Seller, Ainhoa Larumbe, Julio Núñez, Alfonso Valle

**Affiliations:** ^1^Cardiology Department, Hospital Marina-Salud de Denia, 03700 Alicante, Spain; ^2^Cardiology Department, Hospital Clínico Universitario de Valencia, Universidad de Valencia, INCLIVA Health Research Institute, 46010 Valencia, Spain; ^3^CIBER Cardiovascular, Spanish Cardiovascular Research Network, Carlos III Health Research Institute, 28029 Madrid, Spain

**Keywords:** SGLT2 inhibitors, heart failure, acute, admission, congestion, safety

## Abstract

Sodium-glucose co-transporter 2 (SGLT2) inhibitors were initially conceived as glucose-lowering agents. However, striking renal and cardiovascular benefits were observed in type 2 diabetes trials. This led to evaluate it in dedicated studies in chronic heart failure (HF) and chronic kidney disease, which also showed remarkable clinical results. Given this findings, and taking into account the multiple mechanisms of action, the use of SGLT2 inhibitors in acute heart failure seemed promising. Sotagliflozin was the first SGLT2 inhibitor to reduce heart failure hospitalizations within the acute setting in the SOLOIST-WHF trial. Only type 2 diabetes patients were included, with a preserved and reduced ejection fraction. In slightly less than half of the cohort, this medication was started when the diuretic therapy was transitioned from intravenous to oral, during the hospital admission. In the rest of the patients, sotagliflozin was started early after discharge. Empagliflozin proved to be safe, well-tolerated, increased diuresis, and reduced a combined clinical endpoint (worsening HF, rehospitalization for HF, or death at 60 days) when administered within the first 24 hours of an acute heart failure hospitalization in the EMPA-RESPONSE-AHF trial. More recently, empagliflozin showed a reduction in a composite primary endpoint of death, heart failure events, and quality of life compared to placebo in the EMPULSE trial. Empagliflozin was started after the initial stabilization phase, but while patients were still admitted and receiving intravenous loop diuretics. Less than half of the patients were diabetic and two-thirds had a left ventricular ejection fraction below 40%. Dapagliflozin is currently being tested in the DAPA ACT HF-TIMI 68 trial, which plans to enroll 2400 patients admitted with acute heart failure and reduced ejection fraction. We envision SGLT2 inhibitors as a useful tool in acute heart failure syndrome given the additive diuretic effect, and minimal impact on blood pressure, kidney function, and electrolytes. Its dosage schedule is simple and can help initiation and tolerance of other medical therapy. However, there is an increased risk of genital infections and euglycaemic ketoacidosis. Notwithstanding, once critically ill and fasting patients are excluded, early administration of SGLT2 inhibitors is safe. This review summarizes the development of SGLT2 inhibitors and the available evidence supporting their use during an acute heart failure admission. We also propose a practical guideline for in-hospital initiation and monitoring.

## 1. A Walk-Through History and Development of SGLT2 Inhibitors

Sodium-glucose co-transporter type 2 (SGLT2) inhibitors constitute a new drug class initially conceived as glucose-lowering agents. However, the unexpected reduction in cardiovascular morbimortality observed in large clinical trials has led them to emerge as a key treatment in patients with cardiovascular disease, especially heart failure (HF), regardless of the patient’s diabetic status [1].

### 1.1 Origin

SGLT2 inhibitors’ origin dates back to the beginning of the 19th century, when in 1835 Laurent-Guillaume de Koninck and Jean Servais Stats discovered phlorizin, a glycoside present in the roots, leaves, and fruits of the apple tree. However, it was not until 1886 that Freiherr Von Mering described its glucosuric and hypoglycaemic properties [2]. A century later, it was shown that the administration of subcutaneous phlorizin improved the glycaemic profile in pancreatectomized rats. However, this finding was unsuitable for clinical development due to phlorizin’s metabolic instability and nonselective SGLT inhibition [3, 4].

Further chemical research to pursue a selective SGLT2 inhibition led to the development of dapagliflozin in 2008. This agent had a longer half-life, high hydrolysis resistance, and a greater affinity for SGLT2 receptors [5]. Dapagliflozin was the first SGLT2 inhibitor approved in the European Union in 2012, and was followed by canagliflozin (2013), empagliflozin (2014), ertugliflozin (2017), and sotagliflozin (2018).

### 1.2 Mechanisms of Action

Under physiological circumstances, the vast majority of the filtered glucose in the glomerulus is reabsorbed by co-transporters SGLT 1 and 2, coupled with sodium. SGLT1 (low-capacity/high-affinity) is expressed in the intestine, heart, and kidney. The contribution of SGLT1 in glucose reabsorption is minimal compared with SGLT2 (low-affinity/high-capacity), expressed in the proximal tubule of the kidney and responsible for the reabsorption of 90% of the filtered glucose [6, 7]. Thus, SGLT2 inhibition leads to glycosuria and natriuresis, the magnitude of which depends on the circulating glucose concentration and kidney function, decreasing in patients without hyperglycaemia and an estimated glomerular filtration rate (eGFR) <45 mL/min/$m^{2}$ [8].

The primary goal in acute heart failure (AHF) is water and sodium removal. Commonly, variable doses of intravenous loop diuretics are employed to achieve euvolemia. When used in combination with loop diuretics, SGLT2 inhibitors increase the amount of 24-hour urinary volume by approximately 500 mL. Although initial natriuresis is considered to play a role during the first days of treatment, it has not been confirmed in clinical studies. There is no long-term increase in urinary sodium excretion because of compensatory sodium reabsorption distal to the *macula densa*. Therefore, osmotic diuresis seems to be the main driver of increased urinary output. Moreover, some small studies suggest that SGLT2 inhibitors bear complementary diuretic properties over traditional loop diuretics, with a predominant tissue decongestion effect rather than an major repercussion on intravascular volume. Prevailing reduction of intersticial volume could lead to better tissue perfusion and renal hemodynamics [9, 10, 11].

In cases of persistent congestion, urine sodium excretion from loop diuretics can be improved by adding thiazide diuretics. However, sequential nephron blockade with loop and thiazide therapy has been associated with an increased risk of worsening kidney function, electrolyte abnormalities, and neurohormonal activation compared to loop diuretic monotherapy. SGLT2 inhibitors may counteract these adverse events through more electrolyte-free water clearance. The addition of SGLT2 inhibitors to intravenous loop diuretic therapy is expected to provide additional osmotic diuresis while minimizing ionic disorders and avoiding activation of the sympathetic nervous system or renin-angiotensin system [12, 13].

On the other hand, metabolic shift towards ketone body production and free fatty acids utilization may be of great importance during hospital admission. By shunting substantial amounts of carbohydrate into urine, glucose oxidation is progressively substituted by lipid oxidation to generate energy. Under conditions of mild hyperketonemia, β-hydroxybutyrate is freely consumed by the heart and oxidized in preference to fatty acids. This fuel selection improves the transduction of oxygen consumption into work efficiency at the mitochondrial level [14]. Besides, preferential oxidation of ketone bodies may decrease the amount of toxic intracellular lipid metabolites, which could improve cardiac steatosis and left ventricular remodeling. Furthermore, treatment with SGLT2 inhibitors leads to an initial rise in haematocrit, explained by a reduction in plasma volume secondary to the induced osmotic diuresis, and an increase in erythropoietin production due to an enhancement in renal blood flow [15]. Hemoconcentration subsequently promotes oxygen release to the tissues, thereby establishing a powerful synergy with the metabolic substrate shift [16].

**Fig. 1.**
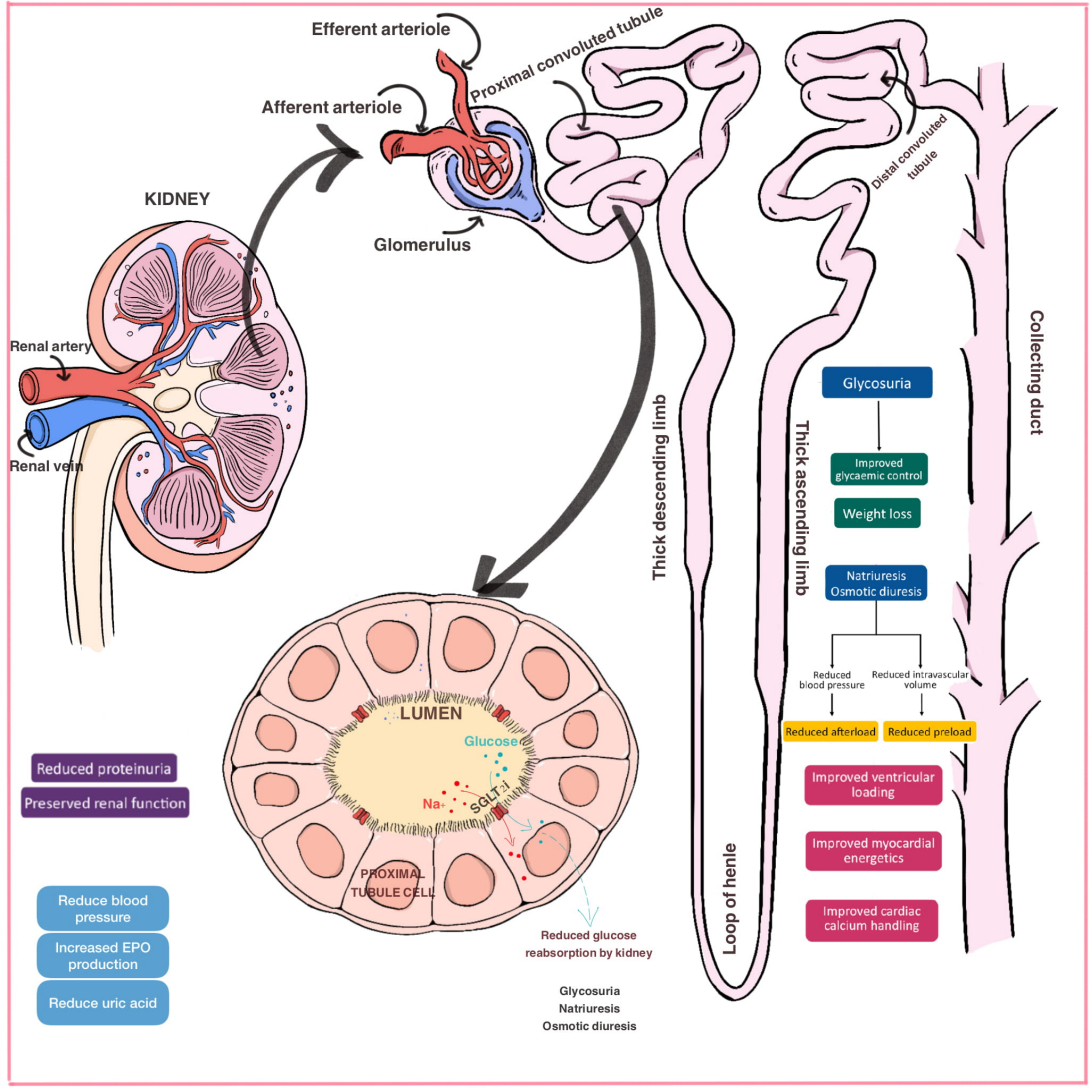
**Mechanisms of action of SGLT2 inhibitors**. Use of SGLT2 inhibitors produces a reduction in glucose reabsorption in the proximal tubule of the kidney, which increases glycosuria and intensifies sodium excretion, promoting an increment in osmotic diuresis. This results in a better glycaemic control, weight loss and blood pressure lowering. On the other hand, shift to fatty substrate utilization improves myocardial energetics and calcium handling. Moreover, SGLT2 inhibitors reduce proteinuria and increase erythropoietin production, which induces a greater renal function preservation over the mid-long term. Abbreviations: EPO, erythropoietin; SGLT2, sodium-glucose co-transporter 2.

Some additional mechanisms of action may be of interest. SGLT2 inhibitors are associated with a mild acute decrease in eGFR secondary to glomerular afferent arteriolar vasoconstriction, which results in a reduction in albuminuria and a better preservation of the renal function over the long-term [17]. Through drug-related glycosuria, SGLT2 inhibitors improve glycaemic control and reduces body mass via calorific loss. A mild decrease in blood pressure and plasma uric acid levels are also observed [18]. Likewise, anti-inflammatory responses, reduction of reactive oxygen species, endothelial function improvement and neuromodulatory effects have been reported. However, their importance is yet to be determined [19, 20, 21, 22]. An overview of SGLT2 inhibitors’ mechanism of action is shown in Fig. 1.

### 1.3 Cardiorenal Benefits in Type 2 Diabetes 

In the last decades, regulatory entities in Europe and United States of America mandated pharmaceutical companies to carry out cardiovascular outcome trials in order to rule out an increase in cardiovascular risk associated with the use of hypoglycaemic drugs. Quite unexpectedly, studies performed with SGLT2 inhibitors in this setting showed a relevant decrease in cardiovascular morbimortality.

This benefit was firstly noted in the Empagliflozin Cardiovascular Outcome Event Trial in Type 2 Diabetes Mellitus Patients (EMPAREG-OUTCOME) trial, in which the use of empagliflozin in diabetic patients with established cardiovascular disease was associated with a significant 14% reduction in the combined endpoint of heart attack, stroke, and cardiovascular death, compared to placebo [23]. Along the same lines, the Canagliflozin Cardiovascular Assessment Study (CANVAS) program demonstrated a 14% reduction in major adverse cardiovascular events and a 33% reduction in hospitalization for HF in diabetic patients with high cardiovascular risk treated with canagliflozin. In addition, the composite outcome of sustained 40% reduction in eGFR, need for renal-replacement therapy, or death from renal causes occurred less frequently among participants in the canagliflozin group [24]. The cardiovascular and renal protection observed in EMPAREG-OUTCOME trial was independent of glycaemic control, suggesting a mechanism of benefit beyond blood glucose-lowering [25].

In the Dapagliflozin Effect on Cardiovascular Events–Thrombolysis in Myocardial Infarction 58** (**DECLARE-TIMI 58) trial, dapagliflozin showed a 17% reduction in the composite endpoint of cardiovascular death and hospitalization for HF in a broad population of patients with type 2 diabetes [26]. On the other hand, ertugliflozin in the Evaluation of Ertugliflozin Efficacy and Safety Cardiovascular Outcomes** (**VERTIS-CV) trial was shown to be non-inferior to placebo in diabetic patients with prior history of cardiovascular disease [27].

More recently, the Effect of Sotagliflozin on Cardiovascular and Renal Events in Patients With Type 2 Diabetes and Moderate Renal Impairment Who Are at Cardiovascular Risk (SCORED) trial enrolled patients with type 2 diabetes and chronic kidney disease. Sotagliflozin, a dual SGLT inhibitor, resulted in a lower risk of the composite of deaths from cardiovascular causes, hospitalizations for HF, and urgent visits for HF, but was associated with an increase in adverse events such as diabetic ketoacidosis, genital mycotic infections, diarrhea and volume depletion [28].

A recent meta-analysis confirmed that SGLT2 inhibitors reduce cardiovascular outcomes in patients with type 2 diabetes mellitus. There was a significant heterogeneity on outcomes between specific agents but a consistent risk reduction in HF hospitalizations, regardless of prior history of atherosclerotic cardiovascular disease or baseline kidney function [29].

### 1.4 Chronic Kidney Disease and Chronic Heart Failure Studies 

#### 1.4.1 Chronic Kidney Disease 

Following these impressive results in type 2 diabetes, trials were carried out to assess the impact of SGLT2 inhibitors in patients with chronic kidney disease (CKD) and chronic heart failure (CHF), irrespective of their diabetic status.

In the Canagliflozin and Renal Events in Diabetes With Established Nephropathy Clinical Evaluation (CREDENCE) trial, the risk of kidney failure and cardiovascular events in patients with type 2 diabetes mellitus and kidney disease was lower in the canagliflozin group than in the placebo group. Moreover, renal protection was independent of glycaemic control [30, 31].

Dapagliflozin was also studied in CKD in the Dapagliflozin And Prevention of Adverse outcomes in Chronic Kidney Disease (DAPA-CKD) trial, both in diabetic and non-diabetic patients. Compared to placebo, dapaglifozin use resulted in a significantly lower risk of a composite of a sustained decline of at least 50% in the eGFR, end-stage kidney disease, or death from renal or cardiovascular causes [32].

#### 1.4.2 Chronic Heart Failure 

In the Dapagliflozin in Patients With Heart Failure and Reduced Ejection Fraction (DAPA-HF) trial, that enrolled 4744 patients with stable HF with reduced ejection fraction (HFrEF), dapagliflozin showed a significant 26% reduction in the risk of cardiovascular death or worsening HF (hospitalization or an urgent hospital visit resulting in intravenous therapy for HF). Additionally, a significant 18% reduction in the risk of cardiovascular death and a similar reduction in all-cause mortality was also reported [33].

In the Empagliflozin Outcome Trial in Patients With Chronic Heart Failure and a Reduced Ejection Fraction (EMPEROR-Reduced) trial, empagliflozin was associated with a 25% lower combined risk of cardiovascular death or hospitalization for HF than placebo, with a slower progressive decline in renal function, regardless of diabetes status. The benefit was primarily related to a 31% reduction in HF hospitalizations. No significant effect in cardiovascular death was observed. Compared to DAPA-HF trial, it enrolled patients with a lower left ventricular ejection fraction (LVEF), higher N-terminal prohormone B-type natriuretic peptide (NT-proBNP) levels and higher use of device and angiotensin receptor-neprilysin inhibitor (ARNI) therapy. However, patients recruited in DAPA-HF trial had a worse New York Heart Association (NYHA) functional class, and a more frequent prior history of hospitalizations due to AHF [34].

Taken together, DAPA-HF and EMPEROR-Reduced trials enrolled patients with a broader spectrum of severity of HF than that of either study alone. A meta-analysis of these two trials concluded that SGLT2 inhibition, when added to optimal medical therapy in patients with HFrEF, reduced all-cause (HR 0.87, 95% CI 0.77–0.98) and cardiovascular death (HR 0.86, 95% CI 0.76–0.98), hospitalizations for HF (HR 0.69, 95% CI 0.62–0.78), and improved renal outcomes. There was no heterogeneity between the two trials and no excess in adverse effects. Results were consistent among subgroups, irrespective of the diabetic status, gender, and ARNI use [35].

Moreover, benefit in worsening HF became apparent in the first days – weeks. Indeed, statistical significance for the primary outcome of cardiovascular death or worsening HF was reached at 28 days after randomization in DAPA-HF trial (HR 0.51, 95% CI 0.28–0.94), and at 12 days after randomization in EMPEROR–Reduced trial (HR 0.42, 95% CI 0.19–0.92) [36, 37]. Dapagliflozin achieved greater relative and absolute risk reductions in those patients with a more recent HF hospitalization. Patients treated with empagliflozin were less likely to require intensification of diuretic therapy, and more frequently experienced an improvement in symptomatic status compared with placebo.

SGLT2 inhibitors showed an early improvement in NYHA functional class and quality of life within three to four months after starting the medication, which was sustained for the rest of the study, both in DAPA-HF and EMPEROR-REDUCED trials. Gain in Kansas City Cardiomyopathy Questionnaire (KCCQ) scores ranged between 1.3 and 2.8 points. More patients on SGLT2 inhibitors had a clinically meaningful (≥5 points) improvement and fewer patients had a ≥5 points deterioration in KCCQ scores, compared to placebo [38, 39].

In addition, SGLT2 inhibitors do not produce clinically relevant changes in blood pressure, renal function, or potassium levels. Specifically, mean systolic blood pressure (SBP) was reduced 1 mmHg with dapagliflozin compared to placebo in DAPA-HF. Even a slight increase in SBP was observed among empagliflozin treated patients in the subgroup of patients with a SBP <110 mmHg at baseline in EMPEROR-Reduced [40, 41]. On the other hand, SGLT2 may cause an initial decrease in eGFR, followed by a slower decline in glomerular filtration rate than placebo, which results in better preservation of renal function in the mid-long term. A sub-analysis of DAPA-HF showed lower rates of hyperkalaemia with dapagliflozin in the subgroup of individuals treated with mineralcorticoid receptor antagonist (MRA). Although this finding was not confirmed in EMPEROR-Reduced, fewer discontinuations of MRA were observed among the empagliflozin treated patients [42, 43].

More recently, the Empagliflozin Outcome Trial in Patients With Chronic Heart Failure With Preserved Ejection Fraction (EMPEROR-Preserved) trial has marked a real turning point in HF. It was the first cardiovascular outcome trial that met its primary endpoint in patients with HF with preserved ejection fraction (HFpEF). Empagliflozin led to a 21% risk reduction of the composite of cardiovascular death or hospitalization for HF compared to placebo, mainly driven by a 29% lower risk of HF admissions [44].

Dapagliflozin, otherwise, showed in the Dapagliflozin Effect on Symptoms and Biomarkers in Patients With Heart Failure (DEFINE-HF) study an improvement in patient-reported symptoms, physical limitations, and exercise function in patients with HFpEF, compared to placebo (clinically meaningful improvement in KCCQ overall summary score or NT-proBNP levels 61.5% vs. 50.4%, adjusted OR 1.8, 95% CI 1.03–3.06) [45]. The Dapagliflozin Evaluation to Improve the LIVEs of Patients With PReserved Ejection Fraction Heart Failure** (**DELIVER) trial, set to determine the impact of dapagliflozin on cardiovascular death, hospitalization for HF, or urgent HF visits in patients with HFpEF, is still ongoing.

Due to its safety, well-tolerability, beneficial cardiac and renal effects in the chronic setting and its variety of different mechanisms of action, early administration of SGLT2 inhibitors during an AHF admission seems attractive.

## 2. Early Initiation of SGLT2 Inhibitors as First-Line Therapy during an Acute Heart Failure Admission

### 2.1 Rationale

AHF is a complex pathophysiological clinical syndrome. The natural history of HF syndrome is progressive, with periods of relative stabilization interspersed with periods of decompensation. AHF refers to rapid or gradual onset of symptoms and/or signs of HF, severe enough for the patient to seek urgent medical attention. AHF is one of the most common causes for hospital admission, with patients hospitalized once a year on average after the initial diagnosis, and is associated with a high risk of mortality. Compared to CHF, there is less robust evidence to guide diagnosis, risk stratification, and management [46, 47, 48].

Heart failure treatment during hospital admission largely relies on clinical expertise and experience. For decades, every study attempting to introduce a new intervention in this field showed neutral or negative findings. Only a few recent studies have presented promising results, indicating that sacubitril-valsartan, omecamtiv mecarbil and ferric carboxymaltose may be appropriate in this challenging clinical setting [49, 50, 51, 52].

On the other hand, the hospitalization period offers clinicians the opportunity to initiate guideline-directed therapies that have been shown to improve long-term morbidity and mortality. It is well known that patients who are not started on neurohormonal medication during admission have less probability of being under treatment with life-saving drugs over the next months [53, 54].

SGLT2 inhibitors have specific properties which may be of great value for AHF syndromes. As previously mentioned, they have proven to reduce HF hospitalizations in stable CHF patients, both in HFrEF and HFpEF, in quick and sustained fashion [33, 34, 44]. At the same time, SGLT2 inhibitors could be of great help in achieving euvolemia during an AHF admission, due to their differential but complementary diuretic effects over traditional loop diuretics. Furthermore, mechanisms of action of these agents are diverse and additive to inhibition of neurohormonal pathways, and could boost in-hospital renin-angiotensin-aldosterone system inhibitors’ prescription and uptitration [8].

Last but not least, administration of SGLT2 inhibitors is simple and does not require special monitoring. It is prescribed as a once-daily single-dose tablet, which does not need further titration. It is usually well tolerated, with a marginal effect on blood pressure and no effect on heart rate, and generally preserves rather than worsens renal function [40, 41, 55, 56].

### 2.2 Scientific Evidence

Sotagliflozin was the first SGLT2 inhibitor to be tested in AHF [57]. The Effect of Sotagliflozin on Cardiovascular Events in Patients With Type 2 Diabetes Post Worsening Heart Failure (SOLOIST-WHF) trial included patients with type 2 diabetes hospitalized with worsening HF, both with HFrEF and HFpEF (ejection fraction <50% in 79% of the patients, respectively). The first dose of sotagliflozin or placebo was administered during the first days after an episode of AHF decompensation. Patients were clinically and hemodynamically stable. Inclusion criteria comprised transition from intravenous to oral diuretic therapy, no need for vasodilator, inotropic nor oxygen therapy, and systolic blood pressure of at least 100 mmHg. Patients had a median age of 70 years, a glycated hemoglobin level of 7.1%, an estimated eGFR of 49.7 mL/min/$m^{2}$, and a median NT-proBNP level of 1800 pg/mL, and could start the study medication during admission (596 patients, 48.8%) or within three days after hospital discharge (626 patients, median two days). Despite early termination because of loss of funding from the sponsor, sotagliflozin reached the primary endpoint of reducing the total number of deaths from cardiovascular causes and hospitalizations and urgent visits from HF at a median follow-up of 9 months (245 vs. 355 events, HR 0.67, 95% CI 0.52–0.85). The benefit was driven by a reduction in HF hospitalizations and urgent visits (40.4% vs. 63.9%, HR 0.64, 95% CI 0.49–0.83), and was consistent among prespecified subgroups stratified according to geographic region, LVEF, timing of the first dose of the medication, sex, age and renal function. As sotagliflozin inhibits both SGLT2 and SGLT1 receptors, diarrhea (6.1% vs. 3.4%) and hypoglycemia (1.5% vs. 0.3%) were more common with sotagliflozin than with placebo.

Some trials regarding SGLT2 inhibition in AHF hospitalized patients have recently been concluded. The Effects of Empagliflozin on Clinical Outcomes in Patients With Acute Decompensated Heart Failure (EMPA-RESPONSE-AHF) pilot study was a randomized, double-blind, placebo-controlled, multicentre trial that enrolled 80 AHF patients with or without diabetes [58]. Patients need to have signs and symptoms of fluid overload, high natriuretic peptides (NT-proBNP ≥1400 pg/mL), and requirements of intravenous loop diuretics. Randomization was performed in the first 24 hours after admission, and treatment was continued through day 30. No difference was observed in any of the primary endpoints, which comprised change in visual analogue scale dyspnea score, diuretic response, change in NT-proBNP, and length of stay. However, empagliflozin was safe, well-tolerated, increased urinary output, and reduced a combined endpoint of worsening HF, rehospitalization for HF, or death at 60 days.

Few small real-life observational studies have confirmed SGLT2 inhibitors’ safety during an AHF admission and emphasize the importance of continuing this antidiabetic drug class at discharge [59, 60]. Two limited sample size randomized trials and three observational studies have suggested an increase in urinary output, total fluid loss, and hemoconcentration with the use of SGLT2 inhibitors during the hospitalization, and a subsequent decrease in congestion and need for loop diuretics at discharge [61, 62, 63, 64, 65].

The biggest evidence supporting the utilization of SGLT2 inhibitors during AHF admission comes from the Empagliflozin in Patients Hospitalized for Acute Heart Failure (EMPULSE) trial, whose results have been recently communicated during the American Heart Association 2021 congress [66]. In this trial, 530 AHF stabilized patients with elevated NT-proBNP (≥1600 pg/mL) requiring at least 40 mg of iv furosemide per day, and eGFR ≥20 mL/min/$m^{2}$ were included. Patients were randomized from day 1 to day 5 after admission, and while still being in the hospital, to empagliflozin 10 mg or placebo. Using a win-ratio approach, empagliflozin significantly reduced the combined primary endpoint of death, the number of HF events, time to first HF event, and change from baseline in KCCQ total symptom score at 90 days (clinical benefit of 53.9% vs. 39.7%, win ratio 1.36, 95% CI 1.09–1.68). Less than half of the patients were diabetic, and two-thirds had a LVEF below 40%. The benefit was consistent across different subgroups and numerically favored empagliflozin for each of the individual components of the primary outcome (death 4.2% vs. 8.3%, HF event 10.6% vs. 14.7%). Body weight was early and steadily reduced with empagliflozin (weight loss of 1.5 kg, present at day 15 after randomization). Serious adverse events were more frequent in the placebo group. Table 1 (Ref. [57, 58, 66]) shows a comparison of SOLOIST-WHF, EMPA-RESPONSE-AHF, and EMPULSE studies.

**Table 1.** **Comparison of SOLOIST-WHF, EMPA-RESPONSE-AHF and EMPULSE trials**.

Trial	Type of SGLT inhibition	Intervention	Main elegibility Criteria	Time of initiation	Follow-up	Primary outcome	Overall treatment effect	Interesting data
SOLOIST-WHF [57]	SGLT1 and SGLT2	Sotagliflozin 200 mg o.d. (uptitrated up to 400 mg) vs. placebo (n = 1222)	Reduced and preserved LVEF	Before discharge (48.8%)	9 months	Total number of CV deaths and hospitalizations and urgent HF visits	51.0 vs. 76.3 events per 100 patient-years	Early termination of the trial because of loss of funding from the sponsor
			Type 2 diabetes	Early after discharge (median 2 days, 51.2%)			HR 0.67 (95% CI 0.52–0.85)	Benefit driven by a reduction in HF hospitalizations and visits
			eGFR ≥30 mL/min/$m^{2}$					Benefit consistent among subgroups and timing of the first dose
								More frequency of diarrhea and severe hypoglycemia in the sotaglifozin group
EMPA-RESPONSE-AHF [58]	SGLT2	Empagliflozin 10 mg o.d. vs. placebo for 30 days (n = 80)	Signs of fluid overload	First 24 hours of admission	60 days	Change in VAS dyspnea score, NT-proBNP, diuretic response and length of stay	Combined mean difference –0.019 (95% CI –0.306–0.269)	No significant difference in any of the primary outcomes
			NT-proBNP ≥1400 pg/mL					Reduction in a combined secondary endpoint of in-hospital worsening HF, rehospitalization for HF or death at 60 days compared with placebo
			Receving loop diuretics					Increase in urinary output up until day 4
								Safety and tolerability. No adverse effects on blood pressure or renal function
EMPULSE [66]	SGLT2	Empagliflozin 10 mg o.d. vs. placebo (n = 530)	NT-proBNP ≥1600 pg/mL	From day 1 to day 5 after admission	90 days	Composite of death, number of HF events, time to first HF event and change in KCCQ-TSS	Clinical benefit 53.9% vs. 39.7%	Numerically relevant reduction in death and HF events with empaglifozin
			Receiving stable ≥40 mg iv furosemide				Win ratio 1.36 (95% CI 1.09–1.68)	Benefit also achieved with standard survival analysis
			eGFR ≥20 mL/min/$m^{2}$					Early and steady weight loss of 1.5 kg
								Serious adverse events more frequent on the placebo group. No cases of ketoacidosis

CI, confidence interval; CV, cardiovascular; eGFR, estimated glomerular filtration rate; EMPA-RESPONSE-AHF, Randomized, double-blind, placebo-controlled, multicentre pilot study on the effects of empagliflozin on clinical outcomes in patients with acute decompensated heart failure; EMPULSE, Empagliflozin in patients hospitalized for acute heart failure; HF, heart failure; HR, hazard ratio; KCCQ-TSS, Kansas City Cardiomyopathy Questionnaire total symptom score; LVEF, left ventricular ejection fraction; NT-proBNP, N-terminal pro hormone B-type natriuretic peptide; o.d., once daily; SGLT, sodium-glucose co-transporter; SOLOIST-WHF, Effect of sotagliflozin on cardiovascular events in patients with type 2 diabetes post worsening heart failure; VAS, visual analogue scale.

Some trials regarding the effect of SGLT2 inhibitors during the acute setting are ongoing. The Dapagliflozin and Effect on Cardiovascular Events in Acute Heart Failure-Thrombolysis in Myocardial Infarction 68 (DAPA ACT HF-TIMI 68) trial will evaluate the effect of in-hospital initiation of dapagliflozin on the clinical outcome of cardiovascular death or worsening HF in a randomized, double-blind, placebo-controlled design. For this purpose, 2400 patients with HFrEF will be enrolled. Study completion is expected for 2023 (ClinicalTrials.gov number, NCT04363697). The effects of early administration of dapagliflozin shortly after discharge will also be evaluated in HFrEF patients with the aim of preventing readmissions and urgent clinic visits of HF (ClinicalTrials.gov number, NCT04249778). In addition, the decongestive effect of dapagliflozin will be tested in a sample of 240 type 2 diabetic patients hospitalized with AHF and presence of congestion. Randomization against placebo on top of a protocolized diuretic therapy will be done within the first 24 hours of presentation to the emergency department (Efficacy and Safety of Dapagliflozin in Acute Heart Failure, DICTATE-AHF trial, ClinicalTrials.gov number, NCT04298229) [67]. Interestingly, A Study to Test Whether Empagliflozin Can Lower the Risk of Heart Failure and Death in People Who Had a Heart Attack (Myocardial Infarction) (EMPACT-MI) trial will evaluate the effect of empagliflozin in an estimated sample size of 5000 patients hospitalized with acute myocardial infarction at high risk of HF. It is a randomized, double-blind, and placebo-controlled study. The treatment will be started during the first 14 days after hospital admission, and the primary outcome is a composite of time to first heart failure hospitalization or all-cause mortality with an expected follow-up of 24 months (ClinicalTrials.gov number, NCT04509674).

### 2.3 Precautions and Risks

As formerly indicated, SGLT2 inhibitors do not induce clinically important modifications in blood pressure, renal function, or potassium levels. During hospital admission, fluid balance and adjustment of diuretic dose can be challenging. However, there was no signal of hypotension or worsening renal failure among empagliflozin groups in EMPA-RESPONSE-AHF or EMPULSE trials [58, 66].

In line with observed in CHF trials, SGLT2 inhibitors were associated with an increase in genital infections within the AHF hospitalization. However, the absolute number of these complications was low, around 1% of treated patients. Nevertheless, women, especially the diabetic and obese, and those with prior history of genital infection are at the highest risk, so counselling and education about personal hygiene and close monitoring and follow-up could be useful in these subgroups [68, 69].

**Fig. 2.**
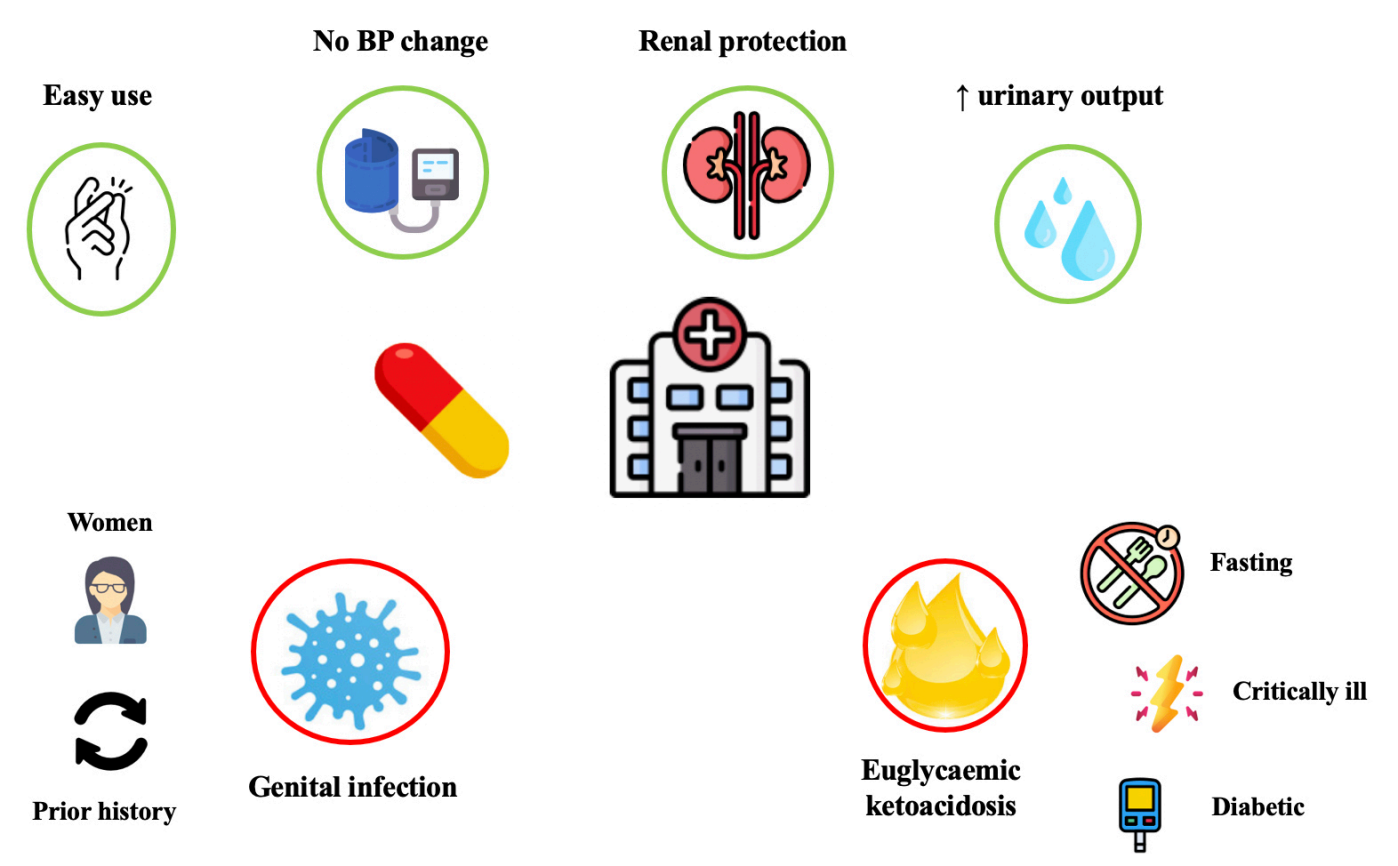
**Benefits and risks associated with in-hospital administration of SGLT2 inhibitors**. Early prescription of SGLT2 inhibitors during an acute heart failure admission is favoured due to its beneficial cardiac and renal properties, easy use and absence of relevant blood pressure or electrolyte changes. However, it may be associated with a small but increased risk of genital infection or euglycaemic ketoacidosis, particularly in certain predisposed patients. Abbreviations: BP, blood pressure.

Nonetheless, the most dreadful secondary effect regarding using SGLT2 inhibitors during an AHF admission is the development of euglycaemic ketoacidosis. Shift to fatty substrate utilization in response to SGLT2 inhibition produces ketones. Rise in ketone levels is usually well-tolerated, but in certain circumstances, like fasting, surgery, infections, or preshock, there may be a meaningful degree of partial insulin deficiency, which enhances the risk of developing ketoacidosis. This increased risk is almost exclusively limited to diabetic patients, especially those treated with insulin or with a low body mass index [70, 71]. Notwithstanding, published data are very reassuring. Among more than 900 patients receiving SGLT2 inhibitors in SOLOIST-WHF, EMPA-RESPONSE-AHF, and EMPULSE trials, only 2 cases of euglycaemic ketoacidosis were described. Moreover, the use of dapagliflozin in non-critically ill COVID-19 hospitalized patients with at least one cardiometabolic risk factor showed only 2 cases of diabetic ketoacidosis (0.3% of the dapagliflozin treated patients). It seems that once critically ill and fasting patients are excluded, prescribing SGLT2 inhibitors in the acute setting is safe and well-tolerated [57, 58, 66, 72]. Fig. 2 summarizes the benefits and risks associated with in-hospital administration of SGLT2 inhibitors.

## 3. Practical Management: When to Start SGLT2 Inhibitors during the Admission

Based on the available evidence, SGLT2 inhibitors are recommended to reduce the risk of HF hospitalization in patients with either established cardiovascular disease or at high cardiovascular risk [73, 74]. Besides, compelling clinical trials have shown their utility in reducing hard outcomes in CHF. In addition, emerging data have confirmed the efficacy and safety of an early introduction of SGLT2 inhibitors in AHF patients, both with and without type 2 diabetes and with reduced and preserved ejection fraction [57, 66].

Results of previous meta-analysis and narrative reviews comprising SGLT2 inhibitors’ effects in the general HF population are in line with current evidence in AHF. A 38% reduction in HF hospitalization was observed grouping 13 randomized clinical trials with more than 14000 patients. This benefit was irrespective of age, gender and diabetes status. Likewise, both cardiovascular and total mortality were significantly reduced. Data regarding SGLT2 inhibitors in AHF showed a clinically relevant, early and sustained reduction in HF admission. To date, no effect in mortality has been consistently demonstrated, possibly due to small sample size of the trials. In previous meta-analysis, adverse events were similar between SGLT2 inhibitors and placebo, except for a mild increase in genital infection in the SGLT2 subgroup. This is concordant with the reassuring safety information regarding SGLT2 inhibitors’ use during the hospitalization [75, 76, 77].

Taking all this data into account, we could hypothesize that the relative benefit of SGLT2 inhibitors on HF outcomes remains constant regardless of target population. As the highest risk patients are those admitted with AHF, the greatest absolute event reduction should be expected in this challenging clinical scenario.

Also, clinical benefit of SGLT2 inhibitors is complementary to neurohormonal medication. The majority of patients included in DAPA-HF and EMPEROR-Reduced trials were treated with at least two of angiotensin-converting enzyme inhibitor (ACEi)/angiotensin receptor blocker (ARB), a betablocker and/or MRA. A small proportion of them were on ARNI. Despite baseline treatment, reduction in the primary outcome was consistent across all subgroups examined, regardless of background therapy or its target doses [78, 79].

**Fig. 3.**
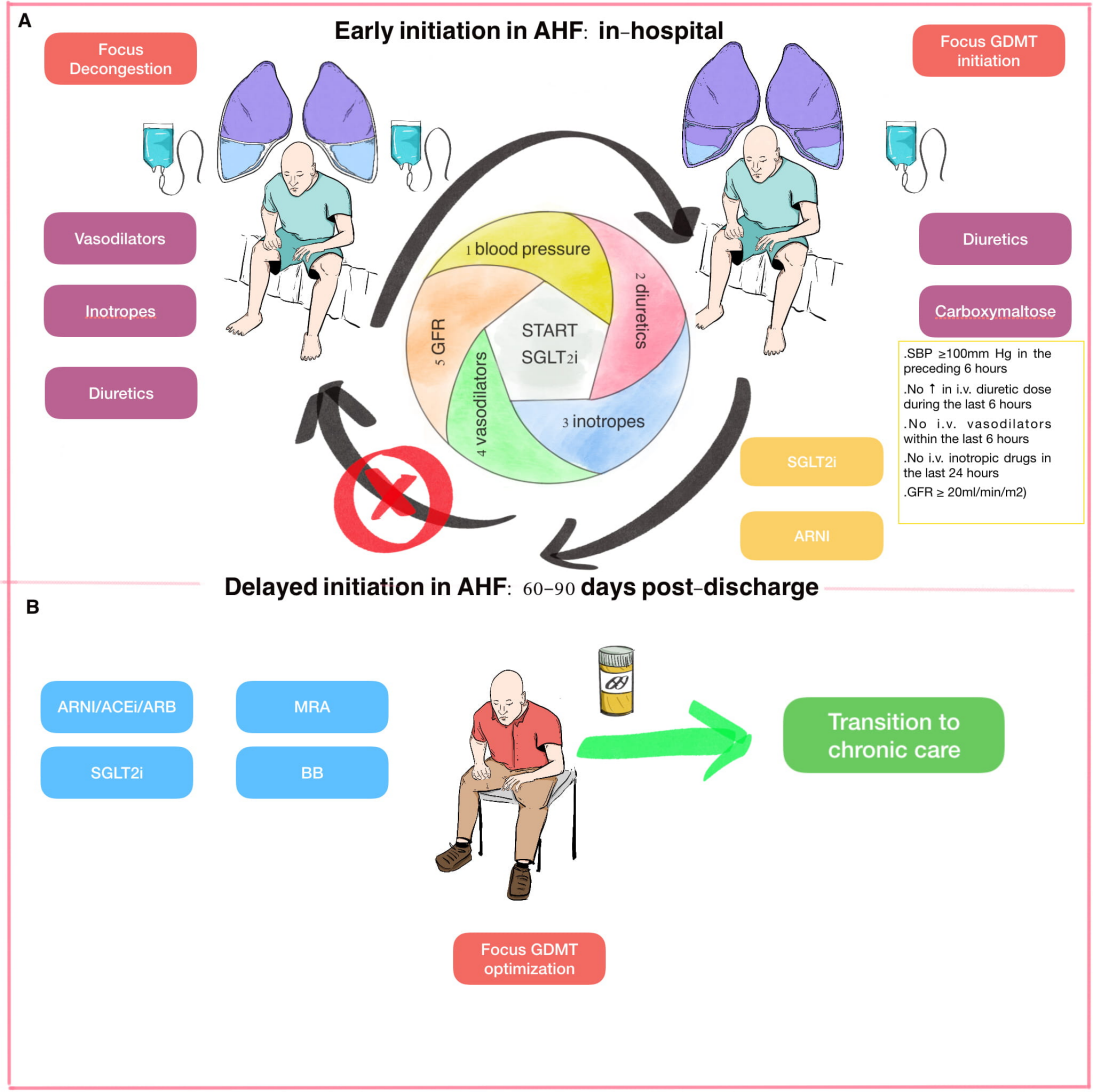
**Early initiation of SGLT2 inhibitors across the continuum of heart failure**. (A) Early phase: during an acute heart failure admission, first early attention is focused on decongestion, usually requiring intravenous treatment (diuretics and/or vasodilators and/or inotropes). It is not recommended to start neurohormonal treatment in these first high-risk hours. (B) Late phase: while the patient’s condition is improving and congestion is decreasing, guideline-directed medical therapy should be initiated, providing some criteria are fulfilled. SGLT2 inhibitors and ARNI have the most compelling evidence in this setting. Abbreviations: ACEi, angiotensin-converting enzyme inhibitor; AHF, acute heart failure; ARB, angiotensin receptor blocker; ARNI, angiotensin receptor-neprilysin inhibitor; BB, beta-blockers; GDMT, guideline-directed medical therapy; GFR, glomerular filtration rate; iv, intravenous; MRA, mineralocorticoid receptor antagonist; SBP, systolic blood pressure; SGLT2, sodium-glucose co-transporter 2.

Before starting SGLT2 inhibitors during an AHF admission, the patient should be clinically and hemodynamically stable and able to tolerate oral intake. A practical guidance scheme is proposed in Fig. 3. Akin to early introduction of ARNI and according to recent clinical trials regarding in-hospital initiation of SGLT2 inhibitors, five criteria have to be fulfilled [50, 66]. First, patients should have a systolic blood pressure above 100 mmHg, and should not have developed any symptoms of hypotension in the preceding 6 hours. Second, progressive and effective decongestion must have been verified, with no need of increasing the intravenous diuretic dose during the last 6 hours. In addition, no prescription of intravenous vasodilators including nitrates within the last 6 hours or administration of intravenous inotropic drugs in the last 24 hours is required. Finally, patients should have a minimally preserved renal function, with an eGFR superior to 20 mL/min/$m^{2}$. Expensive price should also be an issue to bear in mind when prescribing SGLT2 inhibitors.

Although a class effect is plausible and expected, we recommend the use of those SGLT2 inhibitors already tested in HF trials, dapagliflozin and empagliflozin, having the latter greater evidence within the AHF admission. For the time being, sotagliflozin is not currently commercialized.

After prescribing SGLT2 inhibitors, close monitoring of blood pressure, renal function, and urine output is recommended. A subtle decrease in SBP and eGFR and a mild increase in urinary volume are expected. If SBP remains above 90 mmHg, we advise to maintain SGLT2 inhibitors. If SBP drops below 90 mmHg or the patient develop symptoms suggestive of orthostatic hypotension, we advocate for downtitrating the rest of antihypertensive or neurohormonal medication, or reducing diuretic dose if an effective decongestion is being achieved. If SBP remains low despite the previous adjustments, stopping SGLT2 inhibitor should be considered.

Interpretation of renal function changes during the AHF hospitalization is complicated, and should be always made in the context of fluid balance [80]. In general, mild to moderate increases in creatinine (up to 25% of baseline values) are acceptable, and SGLT2 inhibitors should be continued. Even higher transient impairments of renal function may be admissible, especially if a good diuretic response and effective decongestion are taking place. On the contrary, significant deterioration of eGFR due to hypoperfusion, refractory congestion or concomitant use of nephrotoxic drugs is worrisome; an interruption of SGLT2 inhibitors together with a concomitant search for an underlying cause should be carried out.

If the patient was previously taking an SGLT2 inhibitor, it should be continued during the hospitalization, unless presence of severe hypotension or shock. Patients at heightened risk of genital infections or euglycaemic ketoacidosis should be instructed about self-care, prevention, and alarm signs of these complications. In these latter challenging scenarios, further experiences are necessary.

## 4. Conclusions

AHF is a frequent cause of emergency care and hospital admission. It is also associated with high risk in-hospital mortality and short-term rehospitalization. Therefore, therapeutic optimization and early treatment with disease-modifying drugs are a key-issue.

Current available evidence from SOLOIST-WHF, EMPA-RESPONSE-AHF, and EMPULSE trials demonstrate reassuring efficacy and safety data of early introduction of SGLT2 inhibitors during an AHF admission. In addition, SGLT2 have some characteristics of special interest within the acute setting, such as easy use, and absence of relevant blood pressure, kidney function or electrolyte changes. Lastly, the early use of these agents may facilitate the initiation and tolerance of other guideline-directed medical therapy.

While we eagerly await the results of ongoing trials (DAPA ACT HF-TIMI 68, DICTATE-AHF, EMPACT-MI), we recommend starting SGLT2 inhibitors during an AHF admission as soon as an adequate initial response to diuretic, vasodilator and/or inotropic treatment has been checked and the patient can tolerate oral food.

## Abbreviations

ACEi, angiotensin-converting enzyme inhibitor; AHF, acute heart failure; ARB, angiotensin receptor blocker; ARNI, angiotensin receptor-neprilysin inhibitor; CHF, chronic heart failure; CKD, chronic kidney disease; eGFR, estimated glomerular filtration rate; HF, heart failure; HFrEF, heart failure with reduced ejection fraction; HFpEF, heart failure with preserved ejection fraction; KCCQ, Kansas City Cardiomyopathy Questionnaire; LVEF, left ventricular ejection fraction; MRA, mineralcorticoid receptor antagonist; NT-proBNP, N-terminal prohormone B-type natriuretic peptide; NYHA, New York Heart Association; SBP, systolic blood pressure; SGLT2, Sodium-glucose co-transporter 2.
